# Mismatch Repair Protein Expression and Microsatellite Instability in Cutaneous Squamous Cell Carcinoma

**DOI:** 10.3390/curroncol28050287

**Published:** 2021-08-27

**Authors:** Thilo Gambichler, Nomun Ganjuur, Andrea Tannapfel, Markus Vogt, Lisa Scholl, Nessr Abu Rached, Stefanie Bruckmüller, Marina Skrygan, Jürgen C. Becker, Heiko U. Käfferlein, Thomas Brüning, Kerstin Lang

**Affiliations:** 1 Skin Cancer Center, Department of Dermatology, Ruhr-University Bochum, 44791 Bochum, Germany; nomun.ganjuur@klinikum-bochum.de (N.G.); lisa.scholl@klinikum-bochum.de (L.S.); nessr.aburached@klinikum-bochum.de (N.A.R.); stefanie.bruckmueller@klinikum-bochum.de (S.B.); marina.skrygan@klinikum-bochum.de (M.S.); 2 Institute of Pathology, Ruhr-University Bochum, 44789 Bochum, Germany; andrea.tannapfel@rub.de (A.T.); markus.vogt@rub.de (M.V.); 3 Translational Skin Cancer Research, German Cancer Consortium (DKTK) Partner Site Essen/Düsseldorf, Department of Dermatology, University Duisburg-Essen, 47057 Essen, Germany; j.becker@dkfz-heidelberg.de; 4 Translational Skin Cancer Research, DKTK Site Essen, ED03, Deutsches Krebsforschungszentrum (DKFZ), 69120 Heidelberg, Germany; 5 Institute for Prevention and Occupational Medicine of the German Social Accident Insurances, Ruhr-University Bochum (IPA), 44789 Bochum, Germany; kaefferlein@ipa-dguv.de (H.U.K.); bruening@ipa-dguv.de (T.B.); lang@ipa-dguv.de (K.L.)

**Keywords:** non-melanoma skin cancer, cutaneous squamous cell carcinoma, actinic keratosis, mismatch repair deficiency, microsatellite instability

## Abstract

There exist relatively sparse and conflicting data on high-level microsatellite instability (MSI-H) and deficient mismatch repair (dMMR) in cutaneous malignancies. We aimed to determine the expression profiles of MMR proteins (MSH2, MSH6, MLH1, and PMS2) in different progression stages of cutaneous squamous cell carcinoma (cSCC, 102 patients in total) by immunohistochemistry, and search for MSI-H in patients with low-level MMR or dMMR using multiplex-PCR. Low-level MMR protein expression was observed in five patients: One patient with primary cSCC < 2 mm thickness and low-level MLH1, three patients with primary cSCC > 6 mm (including one with low-level MSH2, as well as MSH6 expression, and two with low-level PMS2), and one patient with a cSCC metastasis showing low-level MSH2 as well as MSH6. Intergroup protein expression analysis revealed that MLH1 and MSH2 expression in actinic keratosis was significantly decreased when compared to Bowen’s disease, cSCC < 2 mm, cSCC > 6 mm, and cSCC metastasis. In cases with low-level MMR, we performed MSI-H tests revealing three cases with MSI-H and one with low-level MSI-L. We found low-level MMR expression in a small subset of patients with invasive or metastatic cSCC. Hence, loss of MMR expression may be associated with tumour progression in a small subgroup of patients with non-melanoma skin cancer.

## 1. Introduction

Non-melanoma skin cancer (NMSC) is one of the most common human cancers, with steadily rising incidences. The main subtypes of NMSC, basal cell carcinoma and cutaneous squamous cell carcinoma (cSCC), account for about 99% of all NMSCs [1]. In most cases, cSCC can be curatively managed by means of surgery. However, advanced cSCC, which cannot be treated by surgery and/or radiotherapy, may be a life-threatening condition [2]. Recently, a novel treatment approach with the immune checkpoint inhibitor (ICI) cemiplimab, a potent monoclonal antibody directed against programmed cell death 1 protein (PD-1) receptor, has been approved as monotherapy for adult patients with metastatic or locally advanced cSCC who are not candidates for surgery or radiotherapy. Recent data suggest that about 50% of patients may durably respond to PD-1 inhibitors such as cemiplimab, pembrolizumab, and nivolumab [2,3].

The mismatch repair (MMR) machinery represents an evolutionarily conserved system which is essential for the preservation of cellular DNA homeostasis [4]. This machinery includes the hMutS heterodimers (MSH2/MSH6 and MSH2/MSH3 complexes) ensuring the specific detection of mispaired nucleotides and small insertion-deletion mutations that are formed during replication/recombination processes [2] or caused following DNA damage. The aforementioned hMutS heterodimers are responsible for the initiation of the DNA repair. Moreover, they recruit the hMutL heterodimers (hMLH1/hPMS2, hMLH1/hPMS1, and hMLH1/hMLH3) in order to catalyse the mispair excision and error-free re-synthesis employing the remaining DNA strand as a template for the DNA polymerase [4]. Genetic, as well as epigenetic alterations of MMR genes may result in a hypermutability phenotype that is characterized by spontaneous, genome-wide, mutagenesis [4]. In particular, short tandem repeat DNA sequences, also called microsatellites are affected, making individuals more susceptible to malignancies. In addition to DNA repair, MMR proteins participate in the activation of DNA damage-response pathways that are crucial for protecting against cancer development.

In this context, it is of significance that the prognosis of most malignancies with high-level microsatellite instability (MSI-H) and deficient MMR (dMMR) is relatively good, in particular when treated with ICIs [4]. However, there exist relatively sparse and conflicting data on MSI-H/dMMR in cutaneous malignancies. Several research groups previously investigated MSI-H in NMSC [5,6,7,8,9], also including 56 cSCC. Notably, 53 (56/94.6%) lesions originated from immunosuppressed patients. None of the cSCC assessed in these studies showed MSI-H. The main aim of this study was to determine the expression profiles of MMR proteins in non-immunosuppressed patients with different progression stages of cSCC and search for MSI-H in selected cases with low-level MMR or dMMR.

## 2. Materials and Methods

### 2.1. Patients

We studied 102 patients with, in total, 20 actinic keratoses (AK), 21 Bowen’s disease (BD), 20 invasive cSCC < 2 mm tumour thickness (cSCC < 2 mm), 21 invasive cSCC > 6 mm tumour thickness (cSCC > 6 mm), and 20 cSCC metastases (cSCC-M), (Table 1). All primary tumours were located on the head and neck. cSCC-M consisted of distant skin metastases and four lymph node metastases. Patients with immunosuppression were excluded from the investigation. The study was approved by the ethics review board of the Ruhr-University Bochum (#4749-13) and conducted according to the Declaration of Helsinki principles.

### 2.2. Immunohistochemistry

Tumour sampling was performed using full-thickness excision tissue of the complete lesion. For staining of MLH1, MSH2, MSH6, and PMS2 sections from FFPE blocks (4 µm) were stored for 30 min at 56 °C, deparaffinized in Rotihistol (2 times, 10 min, RT) and then hydrated through graded alcohol series. After heat-induced antigen retrieval for 20 min in EnVision Flex target retrieval solution ‘High pH’ using a steamer, unspecific staining was blocked by incubation in Dako Dual Endogenous Enzyme Block (S2003, Agilent Dako, Hamburg, Germany; 15 min, RT), and additionally 1.5% casein for PMS2 (15 min, RT). Staining was performed using rabbit monoclonal antibodies against PMS2 (M3647) and MSH6 (M3646), and mouse monoclonal antibodies against MLH1 (M3640) and MSH2 (M3639). All antibodies were derived from Agilent Dako. The diluted antibodies against MLH1 (1:50), MSH2 (1:50), and MSH6 (1:50) were incubated for 20 min and against PMS2 (1:40) for 30 min in a humidified chamber at RT. As negative control, sections were incubated without using a primary antibody. The antigen was stained red by the use of the Dako REAL^TM^ Detection System, Alkaline Phosphatase/RED, Rabbit/Mouse (K5005, Agilent) in accordance with the manufacturer’s recommendations, and blue with hematoxylin for nuclear counterstaining. Finally, samples went through a series of ascending alcohol concentrations and were mounted with Entellan (Merck, Darmstadt, Germany). For microscopic analysis, stained slides were scanned at 20× magnification using the Nanozoomer Whole Slide Scanner from Hamamatsu (Hamamatsu, Herrsching am Ammersee, Germany). The images were evaluated by using the viewer software NDP.view2 (Hamamatsu Photonics, Herrsching am Ammersee, Germany).

### 2.3. Microscopic Evaluation

All tumour cells on the entire slide were evaluated. Protein expression was expressed as % of nuclear-stained tumour cells relative to all tumour cells on the slide. In accordance with the College of American Pathologists guidelines for immunohistology evaluation any nuclear tumour cell staining (even patchy) was considered “no loss of expression” and only complete absence of nuclear staining was taken as “loss of expression”, provided that internal controls (e.g., keratinocytes, lymphocytes, and stromal cells) showed nuclear staining [10]. Hence, MMR deficiency was considered when there was complete absence of nuclear staining for at least one protein. Cases with an MMR protein expression of less than 50% were classified as low-level MMR, and cases with an expression of ≥50% as high-level MMR.

### 2.4. Multiplex-PCR and High-Resolution Capillary Electrophoresis

For fragment length analysis, genomic DNA was extracted from neoplastic and corresponding non-neoplastic, microdissected paraffin tissue. Fragment length changes were determined for 8 mononucleotide and dinucleotide markers by multiplex polymerase chain reaction in combination with high-resolution capillary electrophoresis. MSI-H was defined if ≥3 out of 8 markers were found instable, while low-level MSI (MSI-L) was if 1 or 2 markers were instable.

### 2.5. Statistical Analysis

Data analysis was performed using the statistical package MedCalc Software version 15.2 (Ostend, Belgium). Data were analysed using the Chi^2^ test, Kruskal–Wallis ANOVA including the Conover posthoc test for pairwise comparisons, and Spearman or Kendall’s Tau correlation procedures.

## 3. Results

**Figure 1:**
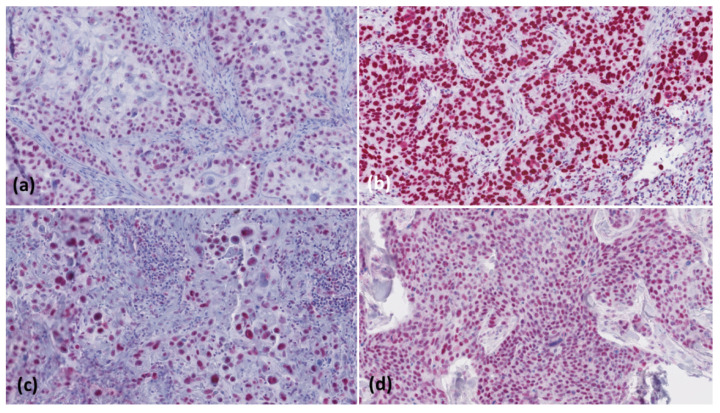
Showing immunohistology images (magnification: ×200) with high-level (≥50%) nuclear expression of mismatch repair proteins; MSH2 (**a**, cSCC > 6 mm), MSH6 (**b**, cSCC > 6 mm), MLH1 (**c**, cSCC < 2 mm), and PMS2 (**d**, cSCC-M).

Together, we studied 102 lesions of patients with a median (range) age of 80 years (47–95). There was no significant difference with respect to age between subgroups (*p* = 0.080). Thirty patients (102/29.4%) were female and 72 (102/70.6%) were male (*p* < 0.0001). The vast majority of tumours investigated showed high-level MMR protein expression (Figure 1).

**Figure 2:**
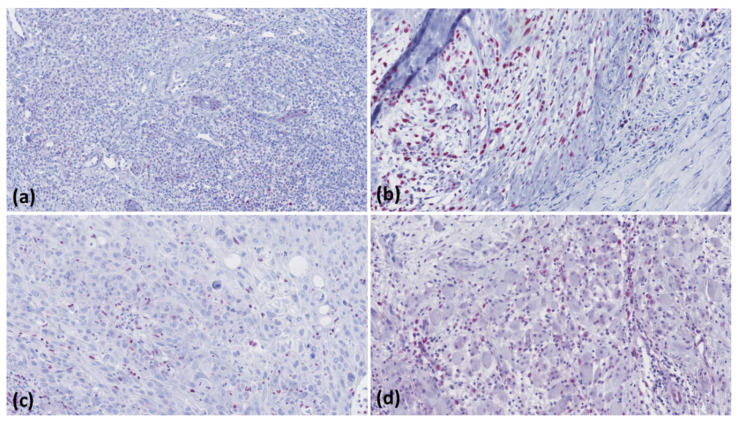
Showing immunohistology images (magnification: ×200) with low-level (<50%) nuclear expression of mismatch repair proteins; MSH2 (**a**, cSCC > 6 mm), MSH6 (**b**, cSCC-M), MLH1 (**c**, cSCC < 2 mm), and PMS2 (**d**, cSCC > 6 mm).

Low-level MMR protein expression was observed in five (102/4.9%) patients (Figure 2), one with cSCC < 2 mm (MLH1 expression: 0.22%), three patients with cSCC > 6 mm (including one with low-level MSH2 (expression: 0.59%) as well as MSH6 (expression: 43.7%) and two with low-level PMS2 (expression: 2.3% and 0.4%)), and one patient with cSCC-M showing low-level MSH2 (expression: 0.64%) as well as MSH6 (expression: 12.1%), (Table 2).

Intergroup protein expression analysis revealed that MLH1 (expression: 98.7%) and MSH2 (expression: 97.4%) in AK was significantly (*p* = 0.014 and *p* = 0.0065, respectively) decreased when compared to BD (expression: 99.5% and 99.5%, respectively), cSCC < 2 mm (expression: 99.2% and 98.8%, respectively), cSCC > 6 mm (99.5% and 99.2%), and cSCC-M (99.4% and 98.6%, respectively). In the cases with low-level MMR, we performed MSI tests revealing three patients with MSI-H, one with MSI-L, and one without MSI.

## 4. Discussion

Reuschenbach et al. [11] studied MSI in 141 epithelial skin lesions, also including 30 cSCC, 41 BD, and one AK. However, none of the skin lesions showed MSI-H at any of the assessed markers [11]. Based on their results and the data reported in the previous studies [5,6,7,8,9], the authors concluded that MSI-H/dMMR is not a relevant tumorigenic mechanism in NMSC [9]. Young et al. [12] found overexpression of MMR proteins in cSCC when compared to normal epidermis. Their analyses also provided evidence for MMR dysregulation in NMSC. However, Young et al. [12] examined nuclear, as well as cytoplasmatic, MMR expression. Muir–Torre syndrome (MTS) and hereditary non-polyposis colorectal cancer (HNPCC) share the same genetic defects in MMR genes and are also associated with different types of NMSC such as keratoacanthoma [13]. Immunohistochemistry for MLH1, MSH2, MSH6, and PMS2 has been reported to be a useful screening method for MTS/HNPCC detection in cases with associated NMSC. Hatta et al. [13] suggested that MSH2 gene and protein abnormalities play an important role in the evolution of skin tumours in addition to the tumours typically included in the MTS diagnostic criteria.

In the present study, we found a tiny but statistically significant decrease in MLH1 and MSH2 expression in AK which was likely caused by difficulties in microscopic evaluation. As the normal keratinocytes also showed strong staining, it was hard to differentiate properly between tumour cells and non-tumour cells. Hence, we do not consider that the statistical difference in MLH1 and MSH2 expression has a true clinical meaning. dMMR per definition (zero expression) was detected in none of the tumour samples [10]. However, we observed low-level MMR expression in about 5% of all cases, which was exclusively detected in invasive or metastatic lesions. MSI-H testing in the five patients with low-level MMR revealed three cases with MSI-H and one with MSI-L. Discordances between MMR and MSI status have previously been reported [10]. Liang et al. [14] previously observed attenuated MSH2 expression in cSCC when compared to AK and BD and suggested that diminished MHS2 expression may occur as a consequence of cancer progression during transformation from pre-malignant epithelial cells into cSCC. MSI-H/dMMR might also be relevant in cutaneous melanoma and its responsiveness to ICI. Korabiowska et al. [15] suggested that, in melanoma, a reduced expression of MMR proteins, rather than a complete loss, is of importance. Interestingly, Ponti et al. [16] studied 14 melanoma patients receiving anti-PD-1 therapy. Using immunohistochemistry for MLH1, MSH2, MSH6, and PMS2, they found that 7% of the tumour samples exhibited dMMR in at least one protein. Three samples from one patient exhibited dMSH6 expression and had the most successful response to anti PD-1 treatment [16]. Recently, we found in nine of 56 (16.1%) patients with Merkel cell carcinoma low-level MMR, whereas MSI-H could be confirmed only in one case [17].

## 5. Conclusions

We found low-level MMR expression and MSI-H/MSI-L in a small subset of patients with invasive or metastatic cSCC. Hence, loss of MMR expression and MSI-H/MSI-L may be associated with tumour progression in a minority of patients with NMSC. Whether these patients have any treatment advantage using ICI must be investigated in future studies.

## Figures and Tables

**Table 1:** One-hundred-and-two non-melanoma skin cancers located on the head and neck excised in non-immunosuppressed patients.

Tumour	Actinic Keratosis	Bowen’s Disease	cSCC < 2 mm Tumour Thickness	cSCC > 6 mm Tumour Thickness	cSCC Metastases
n =	20	21	20	21	20

cSCC = cutaneous squamous cell carcinoma.

**Table 2:** Description of five cutaneous squamous cell carcinoma cases with low-level * mismatch repair protein expression (<50%).

Case#	MLH1	MSH2	MSH6	PMS2
<2 mm	0.22 *	99.45	99.81	68.83
2. >6 mm	99.78	0.59 *	43.67 *	98.81
3. >6 mm	98.69	95.76	99.67	2.32 *
4. >6 mm	97.8	91.19	100	0.42 *
5. metastasis	95.34	0.64 *	12.07	93.85

## Data Availability

Derived data supporting the findings of this study are available from the corresponding author T.G. on reasonable request.
